# From euphoria to wellbeing: Correlates of gender euphoria and its association with mental wellbeing among transgender adults

**DOI:** 10.1080/26895269.2024.2324100

**Published:** 2024-03-08

**Authors:** Ruby Grant, Natalie Amos, Teddy Cook, Ashleigh Lin, Adam Hill, Marina Carman, Adam Bourne

**Affiliations:** a Australian Research Centre in Sex, Health, and Society, La Trobe University, Melbourne, Australia; b ACON Health, Surry Hills, Australia; c School of Population and Global Health, University of Western Australia, Perth, Australia; d Graduate School of Public Health, St Luke’s International University, Tokyo, Japan

**Keywords:** Gender affirmation, gender euphoria, mental health, transgender

## Abstract

**Purpose:**

There is a growing body of literature focusing on the importance of gender euphoria for transgender (hereafter ‘trans’) people. This article aims to explore factors that are associated with experiencing gender euphoria among trans adults in Australia, as well as identifying any correlation between gender euphoria and mental health within this population.

**Methods:**

Data from 1359 trans adults was drawn from *Private Lives 3,* the largest survey of LGBTQ+ people aged 18 and over in Australia. Multivariable logistic regression analyses were conducted to identify the individual factors (sociodemographic characteristics, community connection and access to gender affirming care) associated with having ever experienced gender euphoria and to explore associations between gender euphoria and mental health outcomes, including psychological distress, suicidal ideation and suicide attempt within the past 12 months.

**Results:**

Most (69.1%) of the sample had experienced gender euphoria in their lifetime. However, the likelihood of ever experiencing gender euphoria differed across several factors, including sexual orientation, gender, age, level of education, and residential location. Most notably, non-binary participants were least likely to have experienced gender euphoria. Importantly, participants who felt that they were a part of the LGBTQ+ community in Australia and those who indicated that they were easily able to access gender affirming care were more likely to have experienced gender euphoria. Participants who were currently experiencing gender euphoria were less likely to report high/very high psychological distress and less likely to have experienced suicidal ideation in the past year.

**Conclusion:**

Experiencing gender euphoria may have important implications for the mental wellbeing of trans people, however, some are less likely than others to report these experiences. These findings have important implications for shaping trans affirming practices that enable experiences of gender euphoria.

## Introduction

Gender euphoria is a term used to describe the feelings of happiness and contentment that some transgender (hereafter “trans”) people experience as a result of living in a way that is congruent with their gender identity (Benestad, 2010). This may involve expressing their gender authentically, such as through clothing, hairstyle, or voice, by choosing their name and pronouns, or it may involve seeking gender affirming medical interventions such as hormone therapy or surgeries (Austin et al., 2022; Bradford et al., 2021). While exploration of gender euphoria has been limited in scholarly work until recently, the concept has been explained as “a constellation of feelings related to authenticity, rightness, or “being at home” (Beischel et al., 2022, p. 13). Thus, gender euphoria is considered a positive experience, contributing to an individual’s overall sense of mental health, wellbeing, and quality of life (Beischel et al., 2022). Research has shown that trans people who are able to express their gender in a way that is authentic to them tend to have better mental health outcomes, including lower rates of depression, anxiety, and suicidality (De Vries et al., 2014; Gorin-Lazard et al., 2012; Russell et al., 2018). However, fewer studies have considered associations between gender euphoria and mental health outcomes. Therefore, this article aims to address these gaps by examining the individual factors associated with experiences of gender euphoria and the relationship between gender euphoria and mental health outcomes among trans adults in Australia.

Previous research on trans health has often focused on gender dysphoria rather than gender euphoria. One reason for this is that gender dysphoria, which refers to the distress that some individuals experience because of the incongruence between their gender and their birth registered sex, is a diagnostic criterion for gender incongruence related distress in the DSM-5 (the diagnostic manual used by many mental health professionals) (Austin et al., 2022). As a result, much of the research on trans health has been driven by the goal of understanding and treating this condition (e.g. Reisner et al., 2016). Gender dysphoria can have significant negative impacts on an individual’s mental and physical health, and addressing this distress through gender affirming healthcare has been shown to be an effective way of improving health outcomes and relieving dysphoria (Atkinson & Russell, 2015; Galupo et al., 2020; Morris & Galupo, 2019). Previous research has subsequently interrogated the effectiveness of gender affirming medical interventions in reducing gender dysphoria and improving overall health and wellbeing (Cosio et al., 2022; Gridley et al., 2016; Jarrett et al., 2021). However, experiences of gender euphoria are underexamined in this literature, with studies rarely framing euphoria as a desired, strengths-based, and expansive product of gender affirmation or exploring the links between gender euphoria and other mental health outcomes.

In the emerging body of literature focussing more on the positive aspects of trans experience, gender euphoria is positioned as having the potential to provide both temporary and long-lasting improvements to quality of life for trans people (Austin et al., 2022; Skelton et al., 2023). This work demonstrates how feelings of joy, self-acceptance, optimism, and relief may arise from being recognized and affirmed as one’s gender socially, along with other experiences such as self-acceptance, pleasure, and accomplishment related to gender embodiment and finding community with other trans people (Alutalica, 2021; Jacobsen & Devor, 2022; Shuster & Westbrook, 2022). While some studies have identified gender euphoria as an outcome of medical gender affirmation, or “transition” (Beischel et al., 2022; Skelton et al., 2023), others discourage a medicalised, teleological focus on gender euphoria as a normative conclusion or end point of transitioning (Ashley & Ells, 2018; Shuster & Westbrook, 2022). Rather, gender euphoria has been positioned as a unique and positive aspect of being trans (Alutalica, 2021; Skelton et al., 2023).

Though links between experiences of gender euphoria and improved wellbeing have been established in much of the previous research (Austin et al., 2022; Beischel et al., 2022; Jacobsen & Devor, 2022; Skelton et al., 2023), the majority of this work is qualitative, drawing on smaller samples without examining specific mental health outcomes or demographic factors. One US survey of over 2000 trans adults found that gender euphoria was associated with higher resilience and lower odds of alcohol misuse and gender dysphoria (Reisner et al., 2023). Reisner et al. (2023) also found that trans women and non-binary people were more likely to experience gender euphoria than trans men. However, this study did not find statistically significant associations between gender euphoria and levels of psychological distress, though higher psychological distress was associated with gender dysphoria (Reisner et al., 2023). In this context, it is important to recognize that gender euphoria is not merely the opposite of gender dysphoria, as several qualitative studies have similarly observed distinct experiences of these (e.g. Beischel et al., 2022; Bradford et al., 2021; Hall et al., 2024; Jacobsen & Devor, 2022). Given the importance of gender euphoria in many trans people’s experiences, a deeper understanding of how feelings of euphoria contribute to mental health and wellbeing is necessary. Therefore, in this article, we draw on national data from *Private Lives 3* to identify whether experiences of gender euphoria are associated with better mental health outcomes for trans adults.

## Method

### Sample and procedure

The study’s participants were drawn from *Private Lives 3* (Hill et al., 2020), a nationwide survey, which collected data on the health and wellbeing of 6835 LGBTIQ adults (18 years or older) in Australia. Developed in close consultation with an Expert Advisory Board and a Gender Advisory Board, the survey content and measures used were guided by experts knowledgeable in LGBTQ health and wellbeing in Australia. The survey was accessible for completion from July to October 2019, and participants were recruited *via* paid advertising on Facebook and Instagram and through the outreach efforts of various Australian LGBTQ organizations. The survey encompassed a broad range of topics related to health and wellbeing and included a section specifically related to trans experiences. Only those respondents who indicated that this section was relevant to them were asked to complete it. Participants typically spent between thirty and forty minutes completing the survey. Given the size of the sample, no remuneration or incentive was offered. Ethical approval for the survey was granted by La Trobe University’s Human Research Ethics Committee. For the present article, data were analyzed from 1359 trans participants who also responded to the questions specific to trans people.

## Materials

### Demographics

The survey asked participants about their demographic information, such as age, gender identity, sexual orientation, location of residence, weekly income before taxes, and level of education achieved.

### Experiences of gender euphoria

Participants were asked whether they had ever experienced gender euphoria, with the explanation that gender euphoria refers to “an experience of feeling totally affirmed in one’s gender” (Benestad, 2010). Response options included “No,” “Yes, I am currently experiencing gender euphoria,” “Yes, in the past 12 months,” and “Yes, more than 12 months ago.” For the analyses, responses to this question were either categorized as ever vs never having experienced gender euphoria, or categorized into those that had never experienced euphoria, those that had ever experienced euphoria in the past, and those who were currently experiencing euphoria.

### Feeling a part of the LGBTQ community

Feeling a part of the LGBTQ community was assessed by asking participant the extent to which they agreed with the statement “You feel you’re part of the Australian LGBTIQ community.” Responses were recorded on a 5-point Likert scale ranging from strongly agree to strongly disagree. For the purpose of the analyses, responses were dichotomized into positive (“strongly agree” and “agree”) and not positive (“neither agree nor disagree,” “disagree” and “strongly disagree”). This survey item was adapted and localized from the Connectedness to the LGBT Community Scale (Frost & Meyer, 2012).

### Access to gender affirming care

Being able to access gender affirming care was assessed by asking participants to respond to the item “I have been easily able to access gender affirming care when I have needed to” with responses ranging on a 5-point Likert scale from “Strongly disagree” to “Strongly agree” or the option to choose “Not applicable.” In order to meaningfully interpret the outcomes, responses were dichotomized into positive (“strongly agree” and “agree”) and not positive (“neither agree nor disagree,” “disagree” and “strongly disagree”).

### Psychological distress

Levels of psychological distress were measured using the Kessler Psychological Distress Scale (K10) (Kessler et al., 2002), a 10-item standardized measure addressing symptoms of depression and anxiety as experienced over the previous 4 wk. Participants respond on a 5-point Likert scale ranging from “None of the time” to “All of the time.” Scores can range from 10 to 50, with higher scores indicating higher levels of psychological distress. For the purpose of these analyses, scores were categorized into low or moderate (a score of 10–21) and high or very high (a score of 22–50) psychological distress, based on the thresholds used by the Australian Bureau of Statistics (ABS, 2001).

### Past 12 months suicidal ideation and suicide attempt

Recent experiences of suicidal ideation and suicide attempt were assessed by asking participants if they had experienced any thoughts related to suicide or wanting to end their life as well as if they had attempted suicide or tried to end their life. Response options to these items included “No,” “Yes, in the past 12 months,” “Yes, more than 12 months ago,” and “Prefer not to answer.” Participants were allowed to select multiple responses if they did not select “No” or “Prefer not to answer.” To focus specifically on recent experiences of suicidal ideation and suicide attempt, dichotomous variables were created which indicated whether participants had experienced these in the past 12 months.

## Statistical analysis

All analyses were conducted using Stata (Version 16.1 SE; StataCorp, College Station, TX). Descriptive statistics were used to detail the sociodemographic characteristics of the sample and frequency with which gender euphoria was experienced. Univariable logistic regression analyses were conducted with lifetime experience of gender euphoria as the outcome and each of the sociodemographic factors (sexual orientation, gender, age, income, level of education and residential location), feeling a part of the LGBTQ community in Australia, and ability to access gender affirming care as the independent variables. A multivariable logistic regression analysis was then used to explore factors associated with having ever experienced gender euphoria. Predictor variables included sociodemographic factors (sexual orientation, gender, age, income, level of education and residential location), feeling a part of the LGBTQ community in Australia, and ability to access gender affirming care. To explore the associations between gender euphoria and mental health outcomes, three additional multivariable logistic regression models were conducted, with high/very high psychological distress, lifetime suicidal ideation and lifetime suicide attempt as the outcome variables, and experiences of gender euphoria as the predictor variable. Each of these three regression models additionally controlled for the potential confounding effects of sociodemographic factors as detailed above. No issues with multicollinearity were found with all VIF < 2.0. Unadjusted odds ratios (OR), Adjusted odds ratios (AOR) and 95% confidence intervals (CI), and *p*-values are reported where appropriate.

## Results

A breakdown of the demographic characteristics of the sample are presented in Table 1. More than half of the sample were non-binary (57.7%; *n* = 784), with similar sample size of trans men (21.8%; *n* = 296) and trans women (20.5%; *n* = 279). Participants largely identified as bisexual, pansexual or queer (62.8%; *n* = 849) and were aged between 18-34 years (71.5%; *n* = 972). More than two-thirds (69.1%; *n* = 924) had ever experienced gender euphoria − 47.5% (*n* = 635) reported having experienced euphoria in the past and 21.6% (*n* = 289) were currently experiencing euphoria.

**Table 1.** Sample characteristics (*N* = 1359).

	*n*	%
Gender		
Trans man	296	21.8
Trans woman	279	20.5
Non-binary	784	57.7
Sexual orientation		
Lesbian	149	11.0
Gay	84	6.2
Bisexual	261	19.3
Pansexual	207	15.3
Queer	381	28.2
Asexual	88	6.5
Something else	182	13.5
Age group (years)		
25–34	507	37.3
35–44	465	34.2
45–55	200	14.7
55–64	100	7.4
65–74	87	6.4
Education		
Secondary or below	403	29.7
Non-university tertiary	357	26.3
University-undergraduate	347	25.6
University-postgraduate	251	18.5
Weekly income (pre-tax)		
Nil income	136	10.1
$1–$399	482	35.8
$400–$599	196	14.6
$600–$999	181	13.4
$1000–$1999	273	20.3
$2000+	79	5.9
Residential location		
Capital city, inner suburban	543	40.2
Capital city, outer suburban	401	29.7
Regional city or town	332	24.6
Rural/Remote	75	5.6
Experience of gender euphoria		
Never experienced/unsure	413	30.9
Currently experiencing gender euphoria	289	21.6
Experienced gender euphoria in the past	635	47.5

### Factors associated with ever experiencing euphoria

Several sociodemographic factors were associated with having ever experienced gender euphoria (see Table 2). Compared to lesbian participants, those who identified as asexual had higher odds of ever experiencing gender euphoria (AOR = 2.16, CI = 1.04–4.5, *p* = 0.04). Non-binary participants had lower odds than trans men of experiencing gender euphoria (AOR = 0.65, CI = 0.45–0.92, *p* = 0.017). Compared to those aged 18-24 years, 35-44-year-olds had lower odds of ever experiencing gender euphoria (AOR = 0.56, CI = 0.34–0.93, *p* = 0.023). Participants with an undergraduate university degree had higher odds than those with a secondary school education of having experienced gender euphoria (AOR = 1.71, CI = 1.12–2.62, *p* = 0.014). Finally, participants living in a regional city or town had lower odds of experiencing gender euphoria than those living in an inner-suburban area (AOR = 0.63, CI = 0.44–0.9, *p* = 0.012).

**Table 2.** Factors associated with ever experiencing gender euphoria

	n	%	OR(CI)	P	AOR(CI)	P
**Sexual orientation**						
Lesbian	103	69.1	REF	–	–	–
Gay	46	56.1	0.57 (0.33 – 1.00)	0.049	0.67 (0.32 - 1.41)	0.292
Bisexual	192	75.3	1.36 (0.87 - 2.13)	0.179	1.76 (0.98 - 3.15)	0.059
Pansexual	137	67.2	0.91 (0.58 - 1.44)	0.695	1.20 (0.67 - 2.14)	0.543
Queer	255	68.0	0.95 (0.63 - 1.43)	0.802	1.69 (0.95 – 3.00)	0.074
Asexual	70	79.5	1.74 (0.93 - 3.24)	0.083	2.16 (1.04 - 4.50)	**0.040**
Something else	117	65.7	0.86 (0.54 - 1.36)	0.515	0.98 (0.55 - 1.75)	0.955
**Gender**						
Trans man	212	72.9	REF	–	–	–
Trans woman	225	80.9	1.58 (1.07 - 2.35)	0.023	1.58 (0.95 - 2.64)	0.079
Non-binary	487	63.4	0.65 (0.48 - 0.87)	0.004	0.65 (0.45 - 0.92)	**0.017**
**Age**						
18-24	363	73.0	REF	–	–	–
25-34	308	67.0	0.75 (0.57 - 0.99)	0.040	0.77 (0.52 - 1.14)	0.189
35-44	119	60.4	0.56 (0.40 - 0.80)	0.001	0.56 (0.34 - 0.93)	**0.023**
45-54	66	67.3	0.76 (0.48 - 1.21)	0.252	0.73 (0.39 - 1.38)	0.333
55+	68	80.0	1.48 (0.84 - 2.60)	0.178	0.88 (0.42 - 1.84)	0.726
**Income**						
Nil income	99	73.3	REF	–	–	–
$1 - $399	342	72.5	0.96 (0.62 - 1.47)	0.841	1.07 (0.66 - 1.72)	0.784
$400 - $599	145	75.1	1.10 (0.66 - 1.82)	0.714	1.04 (0.59 - 1.84)	0.886
$600 - $999	112	62.9	0.62 (0.38 - 1.01)	0.053	0.65 (0.37 - 1.13)	0.126
$1,000 - $1,999	163	60.8	0.56 (0.36 - 0.89)	0.013	0.66 (0.38 - 1.16)	0.149
$2,000+	55	69.6	0.83 (0.45 - 1.54)	0.560	1.09 (0.49 - 2.43)	0.824
**Level of education**						
Secondary school	276	69.5	REF	–	–	–
Non-university tertiary	248	71.1	1.08 (0.79 - 1.48)	0.647	1.34 (0.90 - 1.99)	0.151
University-undergraduate	250	72.5	1.15 (0.84 - 1.59)	0.379	1.71 (1.12 - 2.62)	**0.014**
University-postgraduate	150	61.0	0.69 (0.49 - 0.96)	0.026	0.99 (0.61 - 1.61)	0.961
**Residential location**						
Capital city, inner suburban	372	69.5	REF	–	–	–
Capital city, outer suburban	293	74.4	1.27 (0.95 - 1.70)	0.107	1.10 (0.78 - 1.57)	0.589
Regional city or town	209	63.7	0.77 (0.58 - 1.03)	0.078	0.63 (0.44 - 0.90)	**0.012**
Rural/Remote	47	64.4	0.79 (0.47 - 1.32)	0.374	0.63 (0.34 - 1.14)	0.128
**Feel a part of the LGBTQ community in Australia**						
No	313	62.7	REF	–	–	–
Yes	609	72.9	1.6 (1.26 - 2.03)	0.000	1.59 (1.20 - 2.12)	**0.001**
**Easily able to access gender affirming care**						
No	482	67.0	REF	–	–	–
Yes	344	80.4	2.01 (1.51 - 2.68)	0.000	1.83 (1.33 - 2.51)	**0.000**

*Note:* n's and %'s represent the frequency with which participants within each demographic category had ever experienced gender euphoria

Importantly, feeling a part of the LGBTQ community in Australia was associated with higher odds of ever having experienced gender euphoria (AOR = 1.59, CI = 1.2–2.12, *p* = 0.001), as was feeling able to easily access gender affirming care (AOR = 1.76, CI = 0.82–3.79, *p* = 0.149).

### Euphoria and mental health

Compared to participants who had never experienced gender euphoria, those who were currently experiencing euphoria were less likely to report high/very high psychological distress (AOR = 0.42, CI = 0.29–0.61, *p* < 0.001). However, no difference was found between those who had experienced euphoria in the past but not currently, and those who had never experienced euphoria. As with psychological distress, those who were currently experiencing euphoria were less likely to have experienced suicidal ideation in the last 12 months compared to those who had never experienced euphoria (AOR = 0.61, CI = 0.44–0.86, *p* = 0.005), with no difference found between those who had never experienced euphoria and those who had experienced euphoria in the past but were not experiencing it now. No association was found between gender euphoria and having attempted suicide. There are many complex, psycho-social and situational factors, beyond experiences of gender euphoria, contributing to suicide attempts among trans people (removed for peer review), which may override some of the potential benefits that we observed. While a more detailed exploration of the factors influencing trans suicidality is beyond the scope of this paper, our results emphasize the need for ongoing exploration into this critical aspect of trans health and wellbeing (Table 3).

**Table 3.** Associations between experiences of euphoria and mental health outcomes.

	High/very high psychological distress (past 4 weeks)		Suicide ideation (past 12 months)		Suicide attempt (past 12 months)	
	AOR(CI)	*p*	AOR(CI)	*p*	AOR(CI)	*p*
Experience of gender euphoria						
Never experienced/unsure	REF	–	–	–	–	–
Currently experiencing gender euphoria	0.42 (0.29–0.61)	0.000	0.61 (0.44–0.86)	0.005	0.98 (0.54–1.81)	0.959
Experienced gender euphoria in the past	0.79 (0.56–1.12)	0.185	0.91 (0.68–1.22)	0.538	0.92 (0.56–1.49)	0.727

## Discussion

Our findings uniquely reveal associations between gender euphoria and mental wellbeing. Where Reisner et al. (2023) did not find significant associations between psychological distress and gender euphoria, in our sample current experiences of gender euphoria were strongly associated with lower levels of psychological distress and suicidal ideation. Unlike previous research, we noted nuances in the timeframe of when participants had experienced gender euphoria and its association with wellbeing outcomes. For example, no differences in psychological distress were observed between those who had experienced gender euphoria in the past but not currently, and those who had never experienced euphoria. Similarly, those who were currently experiencing euphoria were less likely to have experienced suicidal ideation in the last 12 months compared to those who had never experienced euphoria. Taken together, these findings suggest that current experiences of gender euphoria may be related to improved mental wellbeing for trans people. Given that gender euphoria can be characterized by feelings of happiness, joy, pride, relief, comfort, and self-love (Benestad, 2010), it is perhaps not surprising that such feelings may allay distress and suicidality, even if only in the short term. Conversely, those not experiencing mental ill-health may have more opportunities to experience gender euphoria. As noted in previous research (Beischel et al., 2022; Hall et al., 2024; Jacobsen & Devor, 2022), gender euphoria and gender dysphoria should not be conceptualized as either-or experiences, with experiences of both co-existing at multiple times and levels throughout individuals’ lives. Yet, our findings uniquely suggest that the more immediate the feelings of euphoria, the greater impact they may have on overall wellbeing. This confirms the need to increase opportunities for euphoria through more consistent and ongoing trans affirmation in multiple contexts.

Our study also offers valuable new insights into sociodemographic factors associated with experiences of gender euphoria among trans adults. In contrast to Reisner et al. (2023), we found that non-binary people were less likely to experience gender euphoria than trans men and women. Popular representations and discourse around gender euphoria have largely associated this with experiences of “successfully” conforming to binary gender norms, often through social and medical gender affirmation (Austin et al., 2022). In contrast, non-binary people often report reduced access to medical gender affirmation compared to binary trans people (Kennis et al., 2022). Accordingly, we found that those who had been able to easily access gender affirming medical care were more likely to have experienced gender euphoria. Interestingly, participants who identified as asexual reported high rates of lifetime gender euphoria. As Alutalica (2021, p. 117) outlines, gender euphoria can be a complex component of trans people’s experiences of sex and pleasure because it “highlights the creativity required to feel sexual embodiment when one is navigating sexuality in a gender non-congruent body and exploring means by which sexual expression feels affirmative.” While sex may be a site of complex feelings around gender and embodiment, potentially leading to negative experiences or associations for some trans people, asexual trans people may experience alternative opportunities to explore and express their gender, leading to experiences of euphoria (Sumerau et al., 2018). These is limited research exploring the intersections of sexuality and gender euphoria among trans people, highlighting the need for further exploration. Our results confirm the findings of previous qualitative studies that note the role of community engagement and solidarity in feelings of gender euphoria (Shuster & Westbrook, 2022; Skelton et al., 2023), with those reporting greater LGBTQ community belonging experiencing greater likelihood of experiencing euphoria. This underscores the importance of community support in promoting acceptance, affirmation, and positive mental health and wellbeing for trans adults.

This article makes a necessary quantitative contribution to existing literature by exploring associations between gender euphoria and mental health outcomes, yet it is not without limitations. Notably, we used a convenience sampling methodology, recruiting using targeted social media advertising. As a result, findings may not be representative of all trans people in Australia, though our large sample size potentially minimizes this issue. Because findings were drawn from a survey conducted in 2019, it is important to consider how the impacts of COVID-19 may now produce different outcomes for this population. Given that the data we report here was collected as part of a much larger survey of LGBTIQ+ people in Australia, the questions referring to experiences of gender euphoria were not extensive and further research is required to explore more specific details about trans people’s lived experiences of gender euphoria. Finally, this study was cross-sectional and we are therefore unable to ascertain the direction of associations between gender euphoria and mental health outcomes. Nevertheless, this article is significant in its use of Australia’s largest current sample of trans adults to examine mental health outcomes associated with gender euphoria.

## Conclusion

Experiencing gender euphoria may contribute to greater mental wellbeing for trans people. It is likely that the more immediate the feelings of euphoria, the greater impact they may have on overall wellbeing. These findings demonstrate the need to increase opportunities for gender euphoria through more consistent and ongoing trans affirming practices, such as use of correct name and pronouns, respect for gender expression, and ensuring equitable access to gendered services and spaces. Understanding the benefits of gender euphoria as an aspect of preventative health may assist in further developing approaches to gender affirming care. Increasing our understanding of the role of gender euphoria in the lives of trans people can expand our view of their experiences beyond just gender dysphoria.
